# Simple promotion of Cas9 and Cas12a expression improves gene targeting via an all-in-one strategy

**DOI:** 10.3389/fpls.2024.1360925

**Published:** 2024-03-13

**Authors:** Yiqiu Cheng, Lei Zhang, Jing Li, Xiaofei Dang, Jian-Kang Zhu, Hiroaki Shimada, Daisuke Miki

**Affiliations:** ^1^ Shanghai Center for Plant Stress Biology, CAS Center for Excellence in Molecular Plant Sciences, Chinese Academy of Sciences, Shanghai, China; ^2^ University of Chinese Academy of Sciences, Beijing, China; ^3^ Institute of Advanced Biotechnology and School of Life Sciences, Southern University of Science and Technology, Shenzhen, China; ^4^ Center for Advanced Bioindustry Technologies, Chinese Academy of Agricultural Sciences, Beijing, China; ^5^ Department of Biological Science and Technology, Tokyo University of Science, Tokyo, Japan

**Keywords:** genome engineering, CRISPR/Cas9, CRISPR/ttCas12a, *RPS5a*, all-in-one, gene targeting (GT), transcriptional and translational enhancers

## Abstract

Gene targeting (GT) is a promising tool for precise manipulation of genome sequences, however, GT in seed plants remains a challenging task. The simple and direct way to improve the efficiency of GT via homology-directed repair (HDR) is to increase the frequency of double-strand breaks (DSBs) at target sites in plants. Here we report an all-in-one approach of GT in Arabidopsis by combining a transcriptional and a translational enhancer for the Cas expression. We find that facilitating the expression of Cas9 and Cas12a variant by using enhancers can improve DSB and subsequent knock-in efficiency in the Arabidopsis genome. These results indicate that simply increasing Cas protein expression at specific timings - egg cells and early embryos - can improve the establishment of heritable GTs. This simple approach allows for routine genome engineering in plants.

## 1 Introduction

Gene targeting (GT), such as precise sequence knock-ins (KIs) and substitutions, is a valuable tool for precision genome engineering. Homology-directed repair (HDR)-mediated GT has been used in a variety of organisms, but the extremely low frequency of HDR in seed plants makes GT still a challenging technology (Fauser et al., 2012; Miki et al., 2021a). We recently reported sequential transformation strategies for efficient CRISPR/Cas9-mediated GT in Arabidopsis and rice (Miki et al., 2018; Zhang et al., 2022, 2023; Li et al., 2024). Briefly, in Arabidopsis, the constructs bearing the donor and sgRNA are transformed into parental lines that stably express Cas9 in egg cells and early embryos by the DD45 promoter. Although the efficiency of GT with sequential transformation strategy is higher than with the all-in-one method, the requirement for stable Cas9 transgenic parental lines hinders the broad application of GT in plants, e.g., in cases of different genetic backgrounds. The efficiency of double-strand breaks (DSBs) by sequence-specific nucleases (SSNs) such as Cas9 is one of the most important critical determinants for the efficient establishment of GTs (Zhang et al., 2022; Li et al., 2024). Therefore, the simplest and straight-forward approach to improve the efficiency of GT establishment is to increase the DSB frequency of SSNs. Various approaches have been examined to increase the frequency of DSBs and subsequent GT efficiency. One of these attempts applied the omega translational enhancer from tobacco mosaic virus (TMV) to promote Cas9 translation and successfully improve GT with an all-in-one strategy in Arabidopsis (Peng et al., 2020).

Recently, other transcriptional and translational enhancers have been applied to Cas9 expression to increase the efficiency of mutagenesis in plants. Using the first intron of Arabidopsis *Ubiquitin 10* (AtUbq10) as a transcriptional enhancer, Cas9-mediated heritable mutants were generated at high frequency in barley (Gasparis et al., 2018). Furthermore, dMac3, a highly efficient translational enhancer of the rice *OsMac3* gene, increased the efficiency of targeted mutagenesis by Cas9 and TALEN in rice and potato (Kusano et al., 2018; Onodera et al., 2018; Takeuchi et al., 2021; Kusano et al., 2023; Ohnuma et al., 2023). These enhancers have not been applied much to mutagenesis or GT with the CRISPR/Cas systems, and combinations of enhancers have not yet been reported. To test the utility of the enhancers in establishing GT, the present study used the AtUbq10 transcriptional enhancer and the dMac3 translational enhancer simultaneously, which are expected to drastically improve GT efficiency.

Another difficulty is that the Cas9 sgRNA design limits the target sites of GT. Any coding region or promoter sequence can be targeted if the purpose is to disrupt gene function. On the other hand, pinpoint targeting by SSNs is necessary for GTs such as KI or substitution of nucleotide sequences. The most commonly used *Streptococcus pyogenes* Cas9 (SpCas9: hereafter Cas9) recognizes the NGG (N: A/G/C/T) protospacer adjacent motif (PAM) sequence. If the target sequence of interest is AT-rich or an appropriate sgRNA sequence cannot be designed, this PAM sequence will hinder the broad application of Cas9-mediated GT. Therefore, Cas9 and another popular CRISPR/Cas system, *Lachnospiraceae bacterium ND2006* Cas12a (LbCas12a: hereafter Cas12a), which recognizes TTTV (V: A/G/C) PAM sequences, were applied to Arabidopsis with the aim of establishing GTs at a wider range of target sites. In this study, the temperature tolerant LbCas12a (ttCas12a) variant, which exhibits higher double strand break (DSB) activity under normal growth conditions (22°C) (Schindele and Puchta, 2020), was employed for GT via an all-in-one strategy in Arabidopsis.

In the present study, we investigated a way to improve precise and heritable GT efficiency in an all-in-one method using Arabidopsis as a model. The results show that simply promoting Cas protein expression improves double-strand break (DSB) efficiency, which in turn enhances GT mediated by both Cas9 and ttCas12a in plants.

## 2 Materials and methods

### 2.1 Gene accession numbers

*RPS5A*, At3g11940; *AtUbq10*, At4g05320.

### 2.2 Plant materials and growth condition

The Arabidopsis (*Arabidopsis thaliana*) accession Col-0 was used for all experiments. All plants were grown at 22°C on half Murashige and Skoog (MS) medium or in soil with a 16 h light/8 h dark photoperiod.

### 2.3 Plasmid construction

GT constructs for the all-in-one strategy followed the publications (Miki et al., 2018, 2021a, 2021b). Briefly, a human codon-optimized *Streptococcus pyogenes* Cas9 was used. In addition, ttCas12a was generated by using the human codon-optimized *Lachnospiraceae bacterium ND2006* Cas12a (previously known as LbCpf1) (Wang et al., 2018) and introducing a D156R amino acid substitution (Schindele and Puchta, 2020). For mutation analysis, four constructs with an AtU6-26 promoter-driven sgRNA (or crRNA) cassette and the DD45 promoter and enhancer upstream of Cas proteins were created in pCambia1300. And the *RPS5A*-*Bar* KI donor sequence was cloned into the above plasmids for all-in-one GT. All primers used in this study are listed in **Supplementary Table 1**.

### 2.4 Arabidopsis plant transformation

The generated constructs were transferred to Agrobacterium (*Agrobacterium tumefaciens*) GV3101 competent cells by heat shock method and spread on LB solid medium containing kanamycin and rifampicin, incubated in the dark at 28°C for 2 days to obtain positive transformants. The transformed Agrobacterium is pre-cultured in the 5 mL liquid LB, then grew in a large culture with 150ml LB, and then collected by centrifugation at 4000 rpm for 20 m. The collected Agrobacterium was resuspended in an infection solution containing 5% (w/v) sucrose, 0.22% (w/v) MS and 0.05% (v/v) Silwet-77. Cut off all fruit pods and white flowers from the plants the night before or on the day of transformation. Soak the plant buds in the infection solution for 45 s, remove and shake gently, then wrap in plastic wrap to maintain humidity. The plants were placed in darkness at 22°C for 20 h. The wrapping was removed and the plants were transferred to normal growth conditions.

T1 seeds produced by the flower dipping method were sown on half MS plates containing 50 mg/L hygromycin. Hygromycin-resistant plants were transplanted to soil and screened with three times sprays of Basta at 0.2% (v/v) concentration every three days.

### 2.5 DNA analysis

Genomic DNA was extracted from leaf tissue by the cethyltrimethyl ammonium bromide (CTAB) method for individual plant analysis. Leaf tissues were ground to a fine powder in liquid nitrogen using the ShakeMaster AUTO (Bio Medical Science Inc., Tokyo, Japan). The extracted DNA was used for PCR analysis of GT events. Primers were designed for genotyping and sequencing (**Supplementary Table 1**). The PCR system used 2x Taq Plus Master Mix II (Vazyme, Nanjing, China), according to the instructions. The PCR products were separated by electrophoresis on 1.5% (w/v) agarose gel and visualized by Image Lab software (Bio-Rad Laboratories, Hercules, USA).

The TIDE website (https://tide.nki.nl) was used to determine the mutation frequency of the target *RPS5A* locus (Brinkman et al., 2014). Total DNA was extracted from a pool of three to five independent T1 transgenic plants, and PCR was performed using the extracted total DNA as template. PCR amplicons of the target sites were subjected to Sanger sequencing. For each construct, mutation frequency compared to the Col-0 control was determined. Student’s *t*-test and one-way ANOVA test were performed to compare mutation frequencies between constructs.

To examine the correlation between mutation frequency and GT ratio, the coefficient of determination (*R* ^2^) was calculated.

## 3 Results

### 3.1 Mutation efficiency by Cas9 and ttCas12a using enhancers

**Figure 1:**
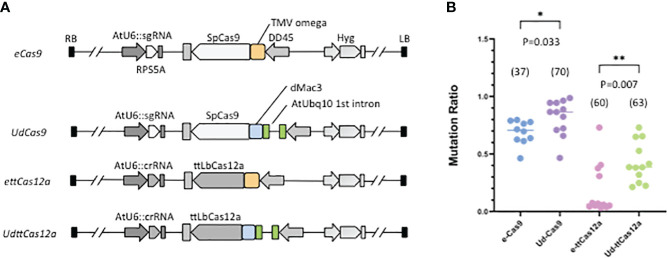
Mutation rates at the target *RPS5A* locus by Cas9 and ttCas12a. **(A)** Schematic diagram of the mutagenesis constructs at the *RPS5A* locus. Pale orange boxes indicate the TMV omega translational enhancer, green boxes represent the AtUbq10 first intron transcriptional enhancer, and blue boxes represent the dMac3 translational enhancer, respectively. All T1 transgenic plants were screened by resistance to hygromycin, followed by PCR and TIDE analysis to determine mutation rates. **(B)** Mutation rates at target *RPS5A* locus in T1 transgenic plants. The numbers in parentheses represent the number of total independent T1 transgenic plants analyzed. The standard deviation of Student’s *t*-test was determined (*P<0.05, **P<0.01).

To examine the contribution of enhancers to DSB, a series of constructs combining the DD45 promoter-driven enhancer Cas and an sgRNA (or crRNA) expression cassette were made. The AtUbq10 first intron was coupled to dMac3 and then linked to Cas9 (*UdCas9*) or ttCas12a (*UdttCas12a*) (**Figure 1A**). The combined use of these two enhancers could be expected to provide much greater improvement than if each enhancer were used alone. The TMV omega translational enhancer was employed as a control (*eCas9*, *ettCas12a*) (**Figure 1A**) because the TMV omega enhancer has been reported to improve Cas9-mediated GT efficiency (Peng et al., 2020). The combination of the two enhancers in this study was expected to highly improve both DSB frequency and GT efficiency over the TMV omega enhancer. The sgRNA and crRNA were designed at the 3’ UTR sequence of *Ribosomal Protein S5 A* (*RPS5A*) (**Supplementary Figure 1**), which is highly expressed in all developmental stages (Tsutsui and Higashiyama, 2017). To assess DSB efficiency, mutation frequencies via the CRISPR/Cas systems were measured. Mutation frequencies were examined in a total of 37 to 70 independent T1 transgenic plants. Total DNA was extracted from a pool of 3 to 5 plants. The target region was amplified using specific primers and subjected to Sanger sequencing. Mutation rates were calculated and determined by the TIDE website. The results showed that the combination of the AtUbq10 and the dMac3 significantly increased the mutation frequency in both Cas9 and ttCas12a compared to the use of the TMV omega enhancer (**Figure 1B**). And the results indicate that the combination of the AtUbq10 enhancer and the dMac3 enhancer improves DSB frequency. Therefore, it was hypothesized that the efficiency of GT would be improved when the enhancers were used in combination for the all-in-one strategy.

### 3.2 Gene targeting via Cas9 and ttCas12a with all-in-one strategy

**Figure 2:**
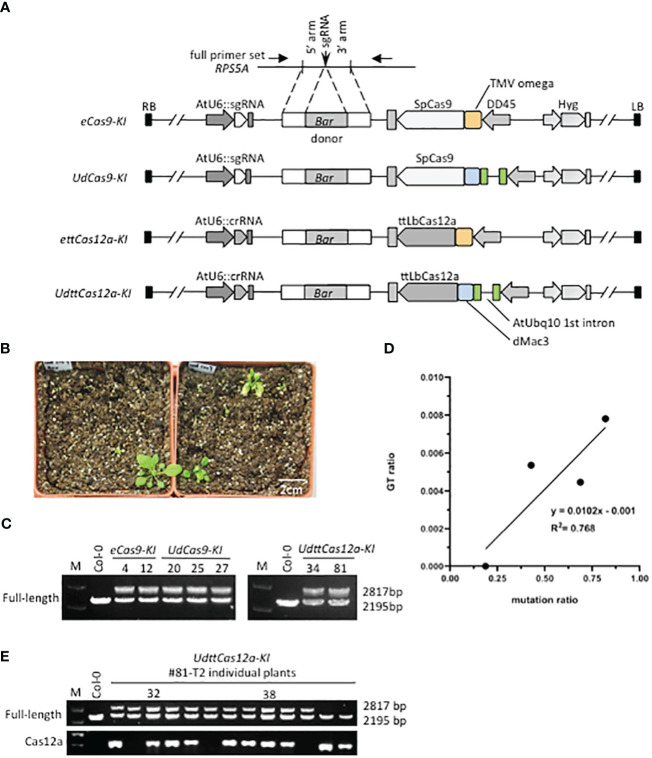
*RPS5A*-*Bar* knock-in gene targeting by Cas9 and ttCas12a. **(A)** Schematic representation of the *RPS5A*-*Bar* KI all-in-one constructs. The KI donor sequences of the 2A and *Bar* gene flanked by 1 Kbp homology arms were cloned into the construct shown in **Figure 1A**. Detailed information is provided in **Supplementary Figure 1**. The full-length primer set used for detecting precise and heritable GT events is indicated by arrows. **(B)** Plant growth after Basta screening. First, Arabidopsis T1 transgenic plants were screened with hygromycin on 1/2 MS plates. Hygromycin-resistant T1 transgenic plants were transplanted to soil and then sprayed with Basta. The white scale bar indicates 2 cm. **(C)** Genotyping *RPS5A*-*Bar* KI in individual T1 plants. The full-length primers were used to detect the precise and heritable GT events. The PCR products with a size of 2195 bp represent the endogenous *RPS5A* and the PCR products with a size of 2817 bp represent the *RPS5A*-*Bar* KI allele. **(D)** Statistical analysis of the relationship between mutation and GT ratio for all four constructs. For statistical analysis, the coefficient of determination (*R* ^2^) between mutation rate and GT efficiency at the *RPS5A* allele in T1 plants was calculated. **(E)** Inheritance of *RPS5A*-*Bar* KI in *UdttCas12a* T2 generation. Precise and heritable GT events were detected by the full-length primer set. The ttCas12a-specific primer set was used to test for the presence of T-DNA.

Four all-in-one KI constructs were designed to determine if the combination of enhancers would improve GT efficiency (**Figure 2A**). In this study, the *RPS5A* gene was chosen as the target of the Basta resistance gene *Bar* KI, and the same sgRNA and crRNA as in the mutation analysis were applied. This is because the *RPS5A* gene is highly constitutively expressed in all developmental and vegetative stages (Weijers et al., 2001; Tsutsui and Higashiyama, 2017). The KI donor constructs consist of the 2A peptide, which functions as a translation initiator for polycistronic mRNA, and the *Bar* gene, flanked by a 1Kbp homology arms (**Figure 2A**, **Supplementary Figure 1**). Thus, it is likely that the precise GT plants will exhibit a strong herbicide Basta resistance phenotype. Two biological repeats were performed for each construct. Transformants were screened with hygromycin on half MS plates, transplanted to soil, and sprayed with Basta (**Figure 2B**). All obtained Basta-resistant plants were subjected to PCR-based genotyping to detect precise and heritable GT events at the target *RPS5A* locus. From 3 to 25 independent Basta resistant T1 transformants were obtained (**Table 1**). Genotyping with full-length primer sets, in which both the precise GT and the endogenous alleles were detectable (**Figure 2A**, **Supplementary Figure 1**), revealed precise GT events in all constructs except *ettCas12a*-*KI* (**Figure 2C**, **Table 1**). GT events detected by full-length primer sets have been reported to accurately incorporate both homologous arms via HDR and to be inherited by progenies (Zhang et al., 2022, 2023; Li et al., 2024). The GT ratio of both Cas9 and ttCas12a was increased with the combination of the enhancers in comparison to the TMV omega enhancer alone (**Figure 2C**, **Table 1**). Statistical analysis revealed a significant positive correlation (*R* ^2^ = 0.768) between mutation and GT ratio (**Figure 2D**). This indicates that DSB frequency by the CRISPR/Cas systems is a crucial factor for the efficiency of GT, a result consistent with the previous reports (Zhang et al., 2022; Li et al., 2024).

**Table 1:** GT efficiencies for RPS5A-Bar KI.

Construct	Enhancer	Hygromycin screening	Basta screening	GT positive	Hygromycin		Basta	
					GT frequency	Average	GT frequency	Average
*eCas9*-*KI*	TMV omega	36	3	0	0%	0.45%	0%	7.14%
		224	14	2	0.89%		14.29%	
*UdCas9*-*KI*	Ubq10 & dMac3	53	4	0	0%	0.78%	0%	9.38%
		192	16	3	1.56%		18.75%	
*ettCas12a*-*KI*	TMV omega	224	25	0	0%	0%	0%	0%
		160	22	0	0%		0%	
*UdttCas12a*-*KI*	Ubq10 & dMac3	224	23	1	0.45%	0.54%	4.35%	6.02%
		160	13	1	0.63%		7.69%	

GT efficiency was calculated based on the number of individual T1 transformants examined.

### 3.3 Inheritance of gene targeting

All precise *Bar*-KI GT events detected by the full-length primer set in the T1 generation were heterozygous and inherited to the next generation as in previous reports (Miki et al., 2018; Zhang et al., 2022; Li et al., 2024) (**Figure 2E**, **Supplementary Figure 2A**). Surprisingly, no homozygous *Bar*-KI GT plants were obtained in the progeny (**Figure 2E**, **Supplementary Figure 2A**). This could be due to lethality caused by RPS5A dysfunction (Weijers et al., 2001). The expression levels of all *RPS5A* mRNAs in *RPS5A*-*Bar* heterozygous T2 plants were similar to those in Col-0 WT plants (**Supplementary Figure 2B**). In this study, the 2A peptide sequence was used to generate two distinct translation products, RPS5A and BAR, from a polycistronic transcript, which often results in a single fusion protein (Barakate et al., 2020). The BAR fusion would have likely interfered with the function of the RPS5A protein. Observations of various semi-dominant phenotypes have been reported in *RPS5A* heterozygous mutants (Weijers et al., 2001). In contrast, *RPS5A*-*Bar* heterozygous GT plants did not show any visible morphological phenotypes. This would be due to the quantity of functional RPS5A protein in the plants. YFP KI at the C-terminal end of the *AFL1* gene has been reported to interfere with the accumulation of AFL1-YFP protein and proper subcellular localization of AFL1 to the membrane (Longkumer et al., 2024). These results suggest that sequence KI to endogenous loci can sometimes hinder their function. Conversely, it is not clear whether RPS5A fusion affects BAR function, which should be investigated in the future.

In addition, *RPS5A*-*Bar* heterozygous T2 plants without transgenes (Cas9 and ttCas12a) were obtained by self-pollination (**Figure 2E**, **Supplementary Figure 2A**). Since the sequential transformation strategy required two backcrosses to remove all transgenes (Zhang et al., 2022), the ability to easily obtain GT plants free of transgenes by self-pollination would be a major advantage of the all-in-one strategy. Furthermore, it has been reported that GT frequency increases in Arabidopsis and barley when donor transgenes are incorporated near endogenous target sites (Fauser et al., 2012; Lawrenson et al., 2021). However, our results suggest that the GT locus and the randomly integrated donor transgenes are not tightly linked in the chromosome (Zhang et al., 2022, 2023).

## 4 Discussion

The sequential transformation strategy, as previously reported, provides a higher efficiency of GT, but requires the use of parental lines, which limits its broad application (Miki et al., 2018, 2021a). Therefore, the establishment of a highly efficient all-in-one GT technology is urgently needed. Here, we demonstrated that the combined placement of enhancers increases the efficiency of GT through both Cas9 and ttCas12a with the all-in-one strategy. Although ttCas12a has been reported to show higher GT efficiency than unmodified Cas12a (Merker et al., 2020), this study demonstrates that GT can be obtained with even higher efficiency by employing enhancers. These results strongly indicate that high levels of Cas protein in egg cells and early embryos efficiently generate DSBs that facilitate homology-directed repair (HDR)-mediated heritable GT establishment in Arabidopsis.

Establishing precise and heritable GTs in seed plants remains difficult due to the extremely low efficiency of homologous recombination (Paszkowski et al., 1988; Fauser et al., 2012). The development of engineered sequence-specific nucleases (SSNs) has facilitated the establishment of GTs in many organisms, but their efficiency in plants is not yet high enough for routine use by universal users (Miki et al., 2021a). Many approaches have been attempted to improve the GT efficiency of plants. The simple and effective way to improve the efficiency of HDR-mediated GT is to promote the efficiency of the DSB. Examples include the use of the highly efficient CRISPR/Cas system (Merker et al., 2020) and the use of enhancers to promote Cas protein expression (Peng et al., 2020). Our sequential transformation method is also one way to promote DSB efficiency. This is because the use of highly efficient parental lines allows maintaining a higher level of Cas9 activity (Miki et al., 2018; Zhang et al., 2022). Transcriptional and translational enhancers have been applied for mutagenesis purposes (Gasparis et al., 2018; Kusano et al., 2018; Onodera et al., 2018; Takeuchi et al., 2021; Kusano et al., 2023; Ohnuma et al., 2023), but rarely for HDR-mediated GT (Peng et al., 2020). During the preparation of this manuscript, it has been reported that the intron-containing version of ttCas12a showed higher GT efficiency than the unmodified ttCas12a (Schindele et al., 2023). These introns may function in the same way as the AtUbq10 first intron in this study, facilitating the transport of mature mRNA into the cytoplasm by splicing and increasing translation efficiency (Mascarenhas et al., 1990; Köhler and Hurt, 2007; Rose et al., 2008). The use of enhancers and introns can increase Cas expression and DSB frequency, resulting in increased GT efficiency. Furthermore, this study showed a strong and statistically significant positive correlation between the DSB ratio and GT efficiency. Taken together, these results indicate that DSB is one of the most important factors determining HDR-mediated GT efficiency.

The objective of this study is to establish a more efficient GT method than previously reported using an all-in-one strategy. With this motivation, the TMV omega enhancer was used as a control in this study to achieve higher GT efficiency. A 3-fold increase in GT efficiency has been reported when using the TMV omega enhancer in the all-in-one strategy (Peng et al., 2020). In this study, a small but significant differences were detected in the enhancer combination, but drastic improvements must be obtained if a version without enhancers is used as a control. Based on previous reports (Gasparis et al., 2018; Kusano et al., 2018; Onodera et al., 2018; Takeuchi et al., 2021; Kusano et al., 2023; Ohnuma et al., 2023), the AtUbq10 first intron and dMac3 is presumed to be an ideal option for improving efficiency, but further analysis is needed, including analysis of individual enhancers separately, as it is also speculated that the combination of enhancers may have some negative effects.

Here, we chose the *RPS5A* gene as a model case for efficient GT establishment. GT plants of *Bar*-KI were expected to exhibit a strong resistance phenotype to Basta herbicide treatments because of the strong and constitutive expression of the target *RPS5A* gene. However, no precise GT events were detected in the majority of Basta-resistant plants. The false antibiotic-positive phenotype of resistance gene KI plants is consistent with previous reports (Wright et al., 2005; Cai et al., 2009; Begemann et al., 2017; Permyakova et al., 2021; Vu et al., 2021). The most likely explanation for the Basta-resistant phenotype in GT-negative plants is the unwanted expression of the *Bar* gene from the randomly incorporated transgenes, even though the 5’ donor homology arm does not contain a promoter sequence. Another possibility is the GT events in which only one homology arm is precisely integrated. A number of GT events have been reported in which one arm is correctly incorporated by HDR and the other arm is T-DNA integrated by NHEJ (Wolter et al., 2018; Wolter and Puchta, 2019; Gao et al., 2020; Huang et al., 2021; Peterson et al., 2021; Zhang et al., 2022; Li et al., 2024). In this study, if the 5’ homology arm is correctly incorporated by HDR into the *PRS5A* locus, both *RPS5A* and *Bar* would be expressed, resulting in a Basta-resistant phenotype. Such GT events in which one arm was precise and the other arm was imprecise were detected fairly frequently (Zhang et al., 2022; Li et al., 2024), but we did not attempt to detect such GT events in the present study. Hence, it is hypothesized that these imprecise GT events contribute to the Basta resistance phenotype.

In this study, Cas9 showed higher DSB ratio and GT efficiency than ttCas12a even though they recognize almost identical target sequence. We consider that these DSB activities are mainly dependent on the design of sgRNAs (crRNAs) and that comparisons of DSB ratio and GT efficiency between Cas9 and Cas12a are irrelevant. Proper design of sgRNAs (crRNAs) and high expression systems of Cas proteins, and high expression of sgRNA also (Li et al., 2024), are crucial to obtain highly efficient and precise GTs. Although only one endogenous target locus was examined in this study, the effects of improvements in the CRISPR/Cas system are usually universal for other loci (Schindele et al., 2023). Therefore, the findings of this study can be widely applied to other plant species as well.

## Data availability statement

The original contributions presented in the study are included in the article/**Supplementary Material**. Further inquiries can be directed to the corresponding author.

## Author contributions

YC: Data curation, Formal Analysis, Investigation, Writing – original draft. LZ: Data curation, Investigation, Writing – review & editing. JL: Data curation, Investigation, Writing – review & editing. XD: Data curation, Investigation, Writing – review & editing. JZ: Funding acquisition, Supervision, Writing – review & editing. HS: Funding acquisition, Resources, Writing – review & editing. DM: Funding acquisition, Investigation, Project administration, Resources, Supervision, Writing – original draft, Writing – review & editing.

## Supplementary material

Supplementary Figure 1 Detailed design of sgRNA and crRNA, *RPS5A*-*Bar* GT constructs and primers. Schematic of the *RPS5A*-*Bar* GT donor construct and the endogenous *RPS5A* locus, showing the full-length primer set used to detect GT events. Red letters for sgRNA and blue letters for crRNA represent PAM sequences, respectively. Green square indicates stop codon. The full-length primers are designed to anneal upstream and downstream of the homology arms and can amplify endogenous and precise knock-in alleles.

Supplementary Figure 2
*RPS5A*-*Bar* KI-GT genotyping and expression in T2. **(A)** Genotyping *RPS5A*-*Bar* KI in *eCas9-KI* and *UdCas9-KI* T2 individual plants. Precise and heritable GT events were detected by the full-length primer set. The Cas9-specific primer set was used to test for the presence of T-DNA. **(B)** qRT-PCR analysis for *RPS5A* expression in T2. For each construct, two heterozygous *RPS5A*-*Bar* GT plants were examined. The primer set for qRT-PCR is designed to anneal upstream the 5’ homology arm and can amplify endogenous and precise knock-in alleles (**Supplementary Figure 1**). The error bars indicate standard deviation of Student’s *t*-test (n=3).

Supplementary Figure 3 Raw data of electrophoresis. Pictures show unprocessed electrophoresis gel images of **Figures 2A, C**, and **Supplementary Figure 2A**.
