# Supplementary material for: Simple promotion of Cas9 and Cas12a expression improves gene targeting via an all-in-one strategy
Source: Front Plant Sci. 2024 Mar 13;15:1360925. doi: 10.3389/fpls.2024.1360925 (PMC10965695; doi:10.3389/fpls.2024.1360925)

Supplementary Table 1. Sequences of primers used in this study.

Primer name and use	Primer sequence (5' – 3')
**sgRNA and crRNA**	
RPS5A-sgRNA	ATACGAGATTGTAACGCACAggg
RPS5A-crRNA	tttcCCTTTGCCCTGTGCGTTACAATC
**Construction**	
RPS5A-5' homology arm-F	TCTTAGATTAGTGTAGAAGATTTGGTAGCTTTCTC
RPS5A-5' homology arm-R	ACGATTAGCCTTGGCAACTCTTTCAATC
RPS5A-3' homology arm-F	TGTGCGTTACAATCTCGTATCAATGAGTTTA
RPS5A-3' homology arm-R	AGGCGGTGGAGAAAATGCACG
Bar-F	ATGAGCCCAGAACGACGC
Bar-R	TCAAATCTCGGTGACGGGC
**GT detection & genotyping**	
RPS5A-FULL-F	CTGGACCTATGACGACGTTACG
RPS5A-FULL-R	GTGTGATGGATGTGCAGATACG
**sequencing**	
RPS5A-2022-F	GAAGATGCTACCAGAATTGGATC

Supplementary Table 2. Raw data of the mutation ratio (Figure 1B).

		mutation ratio	average
e-cas9	1	71.50%	68.80%
	2	63.80%	
	3	79%	
	4	62.60%	
	5	61.30%	
	6	79.60%	
	7	69.70%	
	8	46.30%	
	9	77.80%	
	10	76.40%	
ud-cas9	1	86.60%	82.02%
	2	65.90%	
	3	88.80%	
	4	96.40%	
	5	46.60%	
	6	86.50%	
	7	94.50%	
	8	98.70%	
	9	82.50%	
	10	70.90%	
	11	72.30%	
	12	94.50%	
e-ttcas12	1	40.50%	18.70%
	2	3.90%	
	3	5.20%	
	4	7.50%	
	5	30.80%	
	6	3.30%	
	7	37.60%	
	8	73.10%	
	9	4.40%	
	10	6.90%	
	11	6.00%	
	12	5.20%	
ud-ttcas12	1	21.30%	42.82%
	2	22.40%	
	3	65.50%	
	4	38.20%	
	5	52.80%	
	6	38.50%	
	7	41.50%	
	8	38.60%	
	9	73.10%	
	10	32.00%	
	11	24.90%	
	12	65.00%	

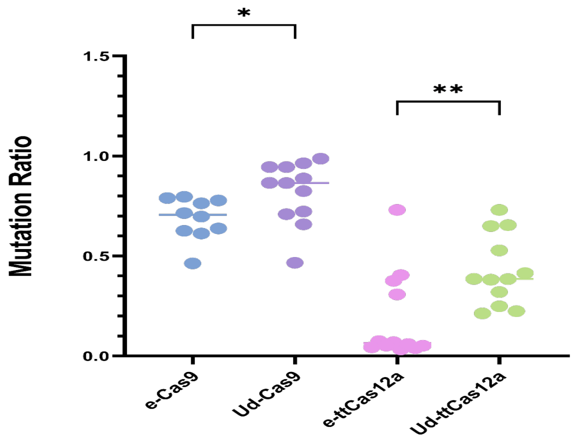

Supplementary Table 3. Raw data for the analysis of the relationship between mutation and GT ratio (Figure 2D).

	mutation	KI
e9	0.688	0.00446
ud9	0.8202	0.007813
e12a	0.187	0
ud12a	0.4282	0.005357

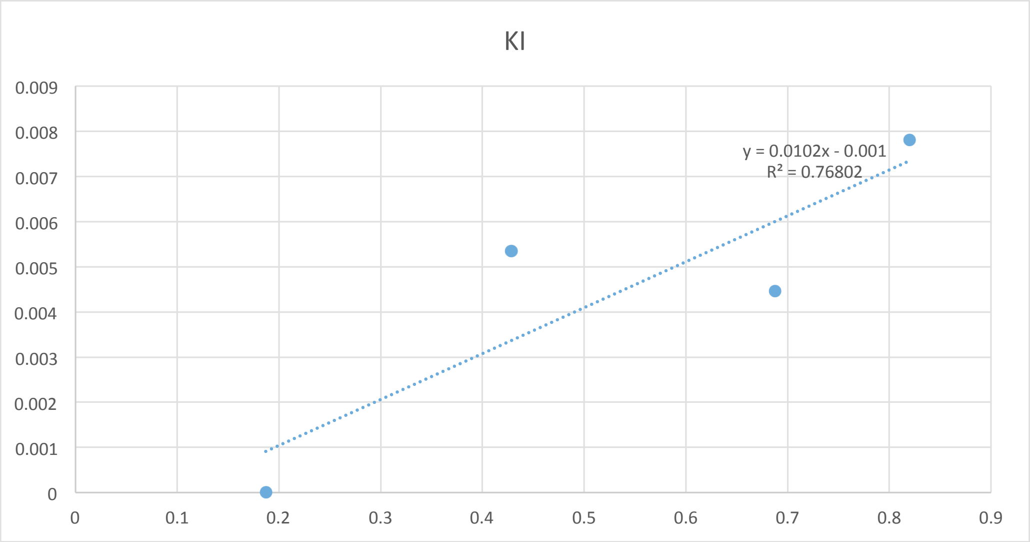

Supplementary Table 4. Raw data for RPS5A expression analysis (Supplementary Figure 2B).

		actin	RPS5A	2^(-△Ct)	average	STDEV-RBCL
	0	26.61378	20.63731	62.96448	67.35597	4.832888
	0	26.60459	20.5478	66.56961		
Col-0	0	26.72853	20.54795	72.53382		
	1	27.7898	21.37158	85.52175	90.82953	5.827781
	1	27.9381	21.44783	89.90101		
	1	28.10773	21.50684	97.06582		
	2	27.16534	21.54668	49.13428	53.35399	3.66607
	2	27.36874	21.56767	55.75646		
eCas9-KI	2	27.23458	21.44874	55.17122		
	20	26.95252	21.14239	56.10815	49.33087	5.985351
	20	26.78264	21.2982	44.76929		
	20	26.91142	21.3533	47.11518		
	22	28.01789	22.26791	53.81658	48.44577	5.5694
	22	27.67259	22.25653	42.69703		
UdCas9-KI	22	27.83994	22.23043	48.82369		
	32	27.31174	21.71081	48.53427	51.40732	4.483069
	32	27.2295	21.61142	49.11462		
	32	27.44367	21.62163	56.57306		
	38	28.1316	22.20937	60.64131	57.25791	5.612562
	38	27.9134	22.24723	50.77922		
UdttCas12-K	38	28.14286	22.2275	60.35321		

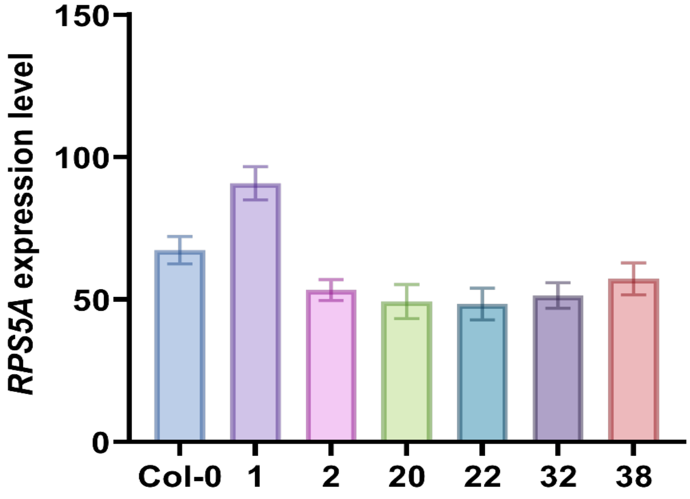

Supplement: Supplementary file 1 [file DataSheet_1.pdf]
