# Supplementary figures and images for: Simple promotion of Cas9 and Cas12a expression improves gene targeting via an all-in-one strategy
Source: Front Plant Sci. 2024 Mar 13;15:1360925. doi: 10.3389/fpls.2024.1360925 (PMC10965695; doi:10.3389/fpls.2024.1360925)

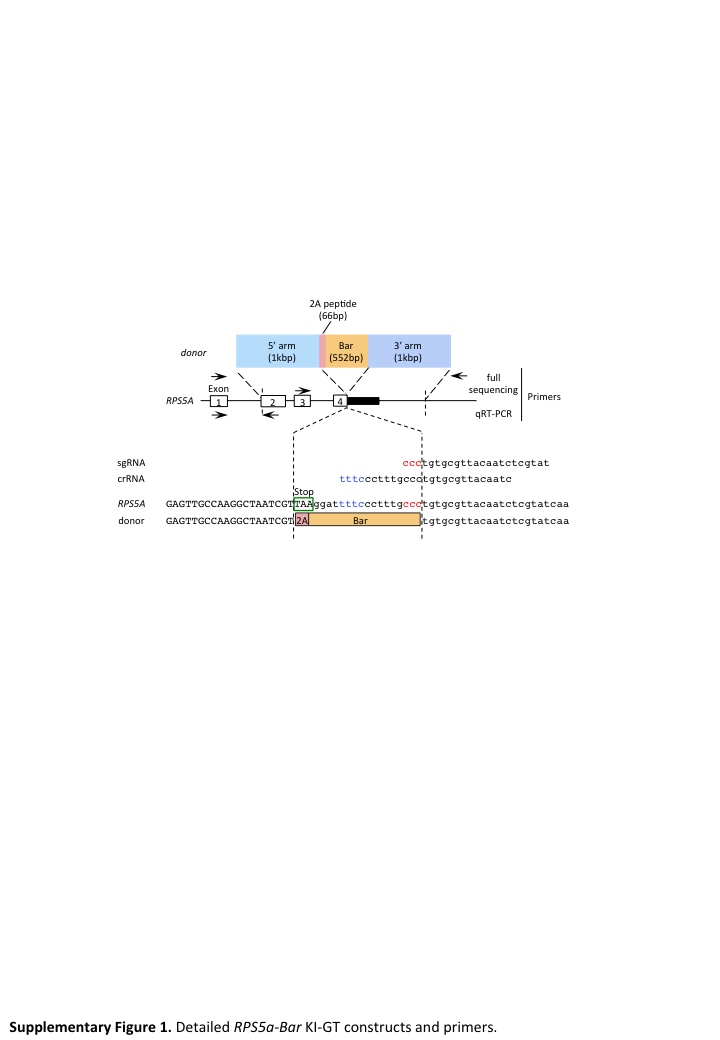

Supplement: Supplementary Figure 1 — Detailed design of sgRNA and crRNA, RPS5A-Bar GT constructs and primers. Schematic of the RPS5A-Bar GT donor construct and the endogenous RPS5A locus, showing the full-length primer set used to detect GT events. Red letters for sgRNA and blue letters for crRNA represent PAM sequences, respectively. Green square indicates stop codon. The full-length primers are designed to anneal upstream and downstream of the homology arms and can amplify endogenous and precise knock-in alleles. [file Image_1.tiff]

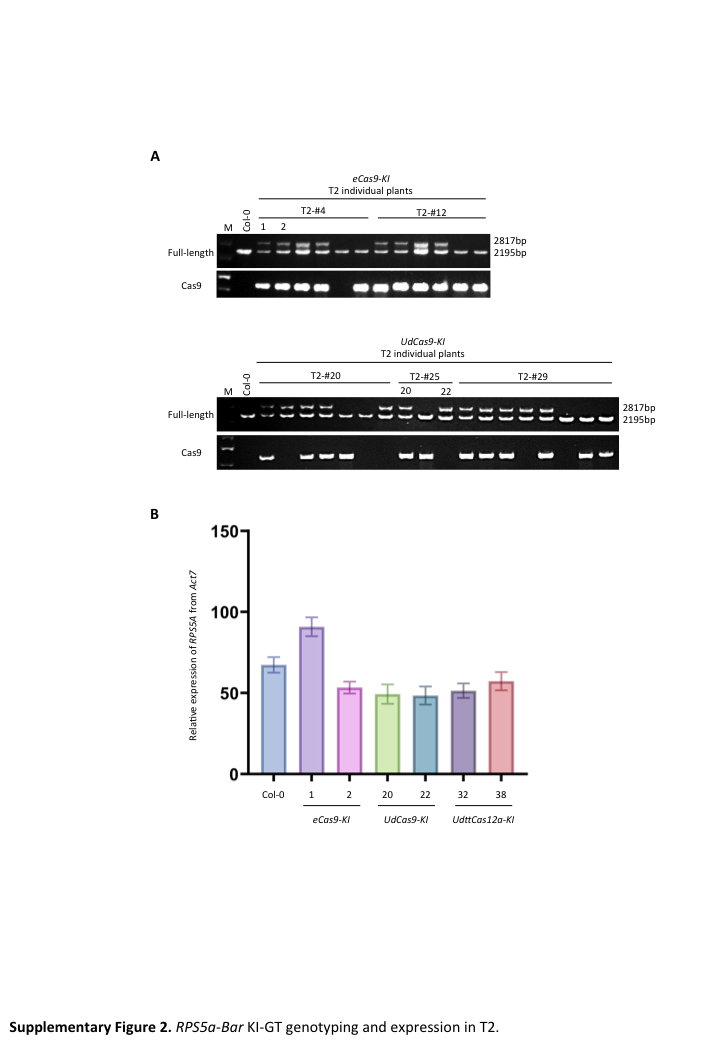

Supplement: Supplementary Figure 2 — RPS5A-Bar KI-GT genotyping and expression in T2. (A) Genotyping RPS5A-Bar KI in eCas9-KI and UdCas9-KI T2 individual plants. Precise and heritable GT events were detected by the full-length primer set. The Cas9-specific primer set was used to test for the presence of T-DNA. (B) qRT-PCR analysis for RPS5A expression in T2. For each construct, two heterozygous RPS5A-Bar GT plants were examined. The primer set for qRT-PCR is designed to anneal upstream the 5’ homology arm and can amplify endogenous and precise knock-in alleles ( Supplementary Figure 1 ). The error bars indicate standard deviation of Student’s t-test (n=3). [file Image_2.tiff]

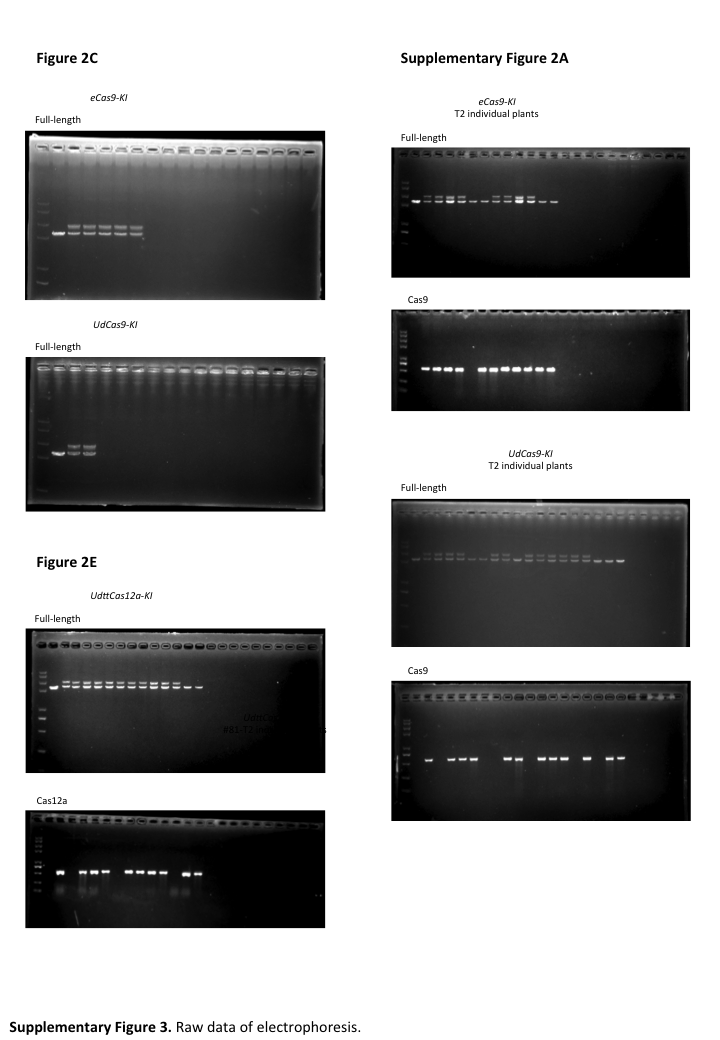

Supplement: Supplementary Figure 3 — Raw data of electrophoresis. Pictures show unprocessed electrophoresis gel images of Figures 2A, C , and Supplementary Figure 2A . [file Image_3.tiff]
